# Metabolomic Impact of Lidocaine on a Triple Negative Breast Cancer Cell Line

**DOI:** 10.3389/fphar.2022.821779

**Published:** 2022-02-22

**Authors:** Thiên-Nga Chamaraux-Tran, Marie Muller, Julien Pottecher, Pierre A. Diemunsch, Catherine Tomasetto, Izzie-Jacques Namer, Nassim Dali-Youcef

**Affiliations:** ^1^ Service d’anesthésie-réanimation et Médecine Périopératoire, Hôpital de Hautepierre, Hôpitaux Universitaires de Strasbourg, Strasbourg, France; ^2^ Institut de Génétique et de Biologie Moléculaire et Cellulaire Illkirch, Illkirch, France; ^3^ Centre National de la Recherche Scientifique, UMR 7104, Illkirch, France; ^4^ Institut National de la Santé et de la Recherche Médicale, U1258, Illkirch, France; ^5^ ER 3072, Mitochondrie Stress Oxydant et Protection Musculaire, Faculté de Médecine, Fédération de Médecine Translationnelle de Strasbourg (FMTS), Strasbourg, France; ^6^ Université de Strasbourg, Faculté de Médecine, Strasbourg, France; ^7^ MNMS-Platform, Hôpital de Hautepierre, Hôpitaux Universitaires de Strasbourg, Strasbourg, France; ^8^ Service de Médecine Nucléaire et d'Imagerie Moléculaire, Institut de Cancérologie Strasbourg Europe, Strasbourg, France; ^9^ ICube, Université de Strasbourg/CNRS, UMR 7357, Strasbourg, France; ^10^ Laboratoire de Biochimie et Biologie Moléculaire, Pôle de Biologie, Hôpitaux Universitaires de Strasbourg, Nouvel Hôpital Civil, 1 Place de l’hôpital, Strasbourg, France

**Keywords:** lidocaine, onco-anesthesia, perioperative period, anesthesia, cancer surgery, metabolomics, cancer progression

## Abstract

**Background:** Metabolomics and onco-anesthesia are two emerging research fields in oncology. Metabolomics (metabolites analysis) is a new diagnostic and prognostic tool that can also be used for predicting the therapeutic or toxic responses to anticancer treatments. Onco-anesthesia studies assess the impact of anesthesia on disease-free and overall survival after cancer surgery. It has been shown that local anesthetics (LA), particularly lidocaine (LIDO), exert antitumor properties both *in vitro* and *in vivo* and may alter the biologic fingerprints of cancer cells. As LA are known to impair mitochondrial bioenergetics and byproducts, the aim of the present study was to assess the impact of LIDO on metabolomic profile of a breast cancer cell line.

**Methods:** Breast cancer MDA-MB-231 cells were exposed for 4 h to 0.5 mM LIDO or vehicle (*n* = 4). The metabolomic fingerprint was characterized by high resolution magic angle spinning NMR spectroscopy (HRMAS). The multivariate technique using the Algorithm to Determine Expected Metabolite Level Alteration (ADEMA) (Cicek et al., PLoS Comput. Biol., 2013, 9, e1002859), based on mutual information to identify expected metabolite level changes with respect to a specific condition, was used to determine the metabolites variations caused by LIDO.

**Results:** LIDO modulates cell metabolites levels. Several pathways, including glutaminolysis, choline, phosphocholine and total choline syntheses were significantly downregulated in the LIDO group.

**Discussion:** This is the first study assessing the impact of LIDO on metabolomic fingerprint of breast cancer cells. Among pathways downregulated by LIDO, many metabolites are reported to be associated with adverse prognosis when present at a high titer in breast cancer patients. These results fit with the antitumor properties of LIDO and suggest its impact on metabolomics profile of cancer cells. These effects of LIDO are of clinical significance because it is widely used for local anesthesia with cutaneous infiltration during percutaneous tumor biopsy. Future *in vitro* and preclinical studies are necessary to assess whether metabolomics analysis requires modification of local anesthetic techniques during tumor biopsy.

## Introduction

In 2020, female breast cancer was the most diagnosed cancer in the world (2,261,419 cases). As almost 685,000 women die of this cancer each year (Sung et al., 2021), breast cancer care is still challenging (Burguin et al., 2021), particularly the triple negative breast cancer (TNBC) subtype which is very aggressive (Bianchini et al., 2021). Customizing care to patient’s phenotypic and/or genotypic background could be an approach to TNBC issues (Burstein et al., 2021; Li et al., 2021).

An emerging strategy to improve survival by personalized medicine and treatment is using metabolomics, an “-omic” approach based on Nuclear Magnetic Resonance (NMR). This technology is an interesting tool for personalized care (Vignoli et al., 2021). Indeed, NMR may provide clues to determine the best therapeutic strategy to follow in patient care and monitoring. High resolution magic angle spinning (HR-MAS) NMR spectroscopy can simultaneously analyze approximately 40 metabolites in biological samples without altering them and can determine tumor metabolomic fingerprints. Many studies have reported a significant association between those fingerprints and clinicopathological status (Choi et al., 2012; Cao et al., 2014; Chae et al., 2016; Tayyari et al., 2018; Vignoli et al., 2021), response to chemotherapy (Cao et al., 2012; Choi et al., 2013) and survival (Giskeødegård et al., 2010, 2012; Cao et al., 2012). Some metabolites are of particular interest: Cao et al. (2012) have demonstrated a significant decrease of glycerophophocholine, phosphocholine, choline and total choline level in survivors in response to treatment compared to non-survivors in breast cancer. Higher levels of glycine and lactate were found to be associated with lower survival rates in breast cancer (Giskeødegård et al., 2012).

Another emerging field of research in cancer care is called onco-anesthesia (Wigmore et al., 2016; Hiller et al., 2017; Cata et al., 2020). Onco-anesthesia investigates the potential impact of anesthesia practices on cancer progression after surgery. Many anesthetic and analgesic drugs used during perioperative period may have a significant impact on immune responses but can also interfere with signaling pathways.

Lidocaine is a commonly used local anesthetics which is often required for local anesthesia before performing fine needle aspiration biopsy or core needle biopsy. It is also employed for regional anesthesia in breast cancer surgery, remote from the surgical site when performing paravertebral block or closer to the wound through plane blocks (pectoral nerves block, serratus blocks, erector spinae plane block … ) (Elshanbary et al., 2021; Gabriel et al., 2021).

In addition to its anesthetic effects, lidocaine can also be administrated intravenously (i.v.) for postoperative analgesia (Beaussier et al., 2018). In breast cancer surgery LIDO contributes to prevent both acute and chronic pain after breast cancer surgery (Grigoras et al., 2012). And it was shown to have anticancer properties (Chamaraux-Tran and Piegeler, 2017; Chamaraux-Tran et al., 2018; Wall and Buggy, 2021; Zhang et al., 2021).

It is also well-know that anesthetic and analgesic drugs do have an impact on cell metabolism (Nouette-Gaulain et al., 2011; Jose et al., 2012). Energy metabolism modulation properties of local anesthetics may stand for a potential therapy to decrease cancer cell proliferation (Jose et al., 2012).

Given the emerging role of metabolomics in breast cancer care, the antitumor properties of local anesthetics and their impact on cell metabolism, we sought to evaluate the impact of lidocaine in metabolomics fingerprints. In an *in vitro* study, we assessed the impact of lidocaine on a triple negative breast cancer human cell line.

## Materials and Methods

### Cells and Cell Culture

MDA-MB-231 (ER and PGR double negative, no amplification of erbB-2 oncogene) human breast cancer cell line representative of the triple negative subtype used throughout this study was obtained from the American Type Culture Collection (ATCC) biological resource center (http://www.atcc.org). The detailed characteristics of the tumor cell line are described elsewhere (Lacroix and Leclercq, 2004). MDA-MB-231 cells were grown in RPMI 1640 medium without HEPES and enriched with 10% fetal calf serum (FCS) and gentamicin (40 μg/ml). Subculturing was routinely carried out every week using diluted trypsin solution (0.25%) in Dulbecco’s phosphate buffered saline (DPBS) without calcium and magnesium (pH 7.2) and cell cultures were kept in a 5% CO_2_ incubator at 37°C.

### Drug Treatment

To perform *in vitro* experiments, lidocaine hydrochloride monohydrate was obtained in a pure lyophilized form (MW 288.81, Sigma-Aldrich, St. Louis, MO). A stock solution (50 mg/ml in H_2_O) was freshly prepared and increasing drug concentrations (0.1, 0.5, 1, 5, 10 mM) were obtained by diluting the stock solution in cell culture medium. Final pH of lidocaine-containing or -free (control) mediums were controlled and were found to be equivalent.

### MTT Assay for Cell Viability

This rapid colorimetric assay using 3-[4,5-dimethylthiazol-2-yl]-2,5-diphenyltetrazolium bromide; thiazolyl blue (MTT) was elaborated by Mosmann *et al.* to assess cellular growth and survival (Mosmann, 1983). Exponentially growing cells were enzymatically detached and a single tumor cell suspension in culture medium at a density of 30 × 10^3^ cells/ml was prepared. Cells were seeded in 24-well microtiter plates (1 ml/well) and allowed to attach for 48 h under the previous specified conditions. Culture medium in each well was aspirated and replaced with fresh culture medium containing the different lidocaine concentrations and allowed to grow for a further 4 h. Triplicate wells were used for controls (H_2_O as vehicle alone) and each concentration of lidocaine. Cell viability was then determined using the MTT assay (Marks et al., 1992) with minor modifications. In brief, 100 µl of MTT (5 mg/ml in DPBS) (3-(4,5 dimethylthiazol-2-yl) 2,5 diphenyl-tetrazolium bromide) were added and the plates were incubated at 37°C for 1 h in the dark. This assay is based on the cleavage of the tetrazolium salt by viable cells and the accumulation of a water insoluble formazan salt proportional to the number of living cells in the well. After careful aspiration of the culture medium, 100 µl of DMSO were added to each well and the plates were incubated for a further 1 h. Absorbances were then measured for each treatment condition at a wavelength of 550 nm with reference to the appropriate blank (DMSO only) in a 96-wells microplate spectrophotometer (ELx808 Absorbance Microplate Reader, Biotek Instruments and Gen5 Data Analysis Software 1.06) and compared to control untreated cells.

### ^1^H-High Resonance Magic Angle Spectroscopy (^1^H-HRMAS) Metabolomic Data Acquisition and Processing

For this experiment, 10^7^ MDA-MB-231 cells were seeded in 750 ml cell culture flask with a polystyrene growth area of 175 cm^2^ for 24 h. Culture medium was then aspirated and replaced with fresh culture medium containing lidocaine (at concentration of 0.5 mM, *n* = 5) or the same volume of H_2_O (*n* = 4). After 4-h incubation at 37°C, medium was removed, and cells were washed by phosphate-buffered saline (PBS 1M). Cells were trypsin-detached and centrifugated at 1,200 rpm to throw supernatant. Cell pellet was then homogenized and 20 µl of the cap was put into a cryotube. Manual centrifugation was performed to remove any air bubbles and the cryotube was immediately placed in liquid nitrogen for rapid freezing. Five microliters of deuterium oxide were added before −20°C storage.

NMR HRMAS data acquisition and processing have been previously detailed (Battini et al., 2016). Briefly, NMR HRMAS assay was performed by 500 MHz Bruker Avance III spectrometer. A 1-dimensional (1D) proton spectrum using a Carr-Purcell-Meiboom-Gill (CPMG) pulse sequence was acquired for each sample with a 285 μs interpulse delay and a 76 min acquisition time for each tissue sample. The number of loops was set at 328, giving the CPMG pulse train a total length of 93 ms. The chemical shift was calibrated to the peak of the methyl proton of L-lactate at 1.33 parts per million (ppm). Unidimensional (1D) acquisition was immediately followed by a 2-dimensional (2D) heteronuclear experiment (in order to confirm resonance assignments). Heteronuclear Single Quantum Coherence (HSQC) spectrum was acquired during 15 hs and 22 mns (time acquisition: 0.073s (1H)/0.006s (13C), 136 scans, spectral window: 7,002 Hz (1H)/20,833 Hz (13C), relaxation time: 1.5 s). Metabolites were assigned using a standard metabolite chemical shift table available in the literature (Martínez-Bisbal et al., 2004; Wishart et al., 2007). Metabolite quantification was performed using an external reference standard of lactate (19,3 nM), scanned under the same analytical conditions. Spectra were normalized according to sample weight. Peaks of interest were automatically defined by an in-house program using MATLAB (MATLAB R2010; MathWorks, Natik, MA).

### Statistical Analysis

#### Data Are Expressed as Mean ± Standard Deviation

MTT *in vitro* assay was performed in triplicate and at least three times. Results were compared with one-way repeated measures ANOVA followed by a Dunnett test. GraphPad InStat statistics software (GraphPad Software, Inc., La Jolla, CA) was used for these analyses. *p* values < .05 were considered statistically significant.

Network analysis was obtained using the Algorithm to Determine Expected Metabolite Level Alterations Using Mutual Information (ADEMA) which has been applied on metabolite quantification values. ADEMA processing has been previously detailed (Cicek et al., 2013; Battini et al., 2016; Bender et al., 2020). Briefly, this method allows for the comprehensive analysis of variations in a pathway of metabolites within cells exposed or not to lidocaine. Instead of analyzing the metabolites one by one, ADEMA integrates them into the topology of the metabolic network that was built according to the Kyoto Encyclopedia of Genes and Genomes (Kanehisa and Goto, 2000) and Salway’s work (Salway, 2016).

## Results

### High Concentration of Lidocaine Impairs Cell Viability

**FIGURE 1:**
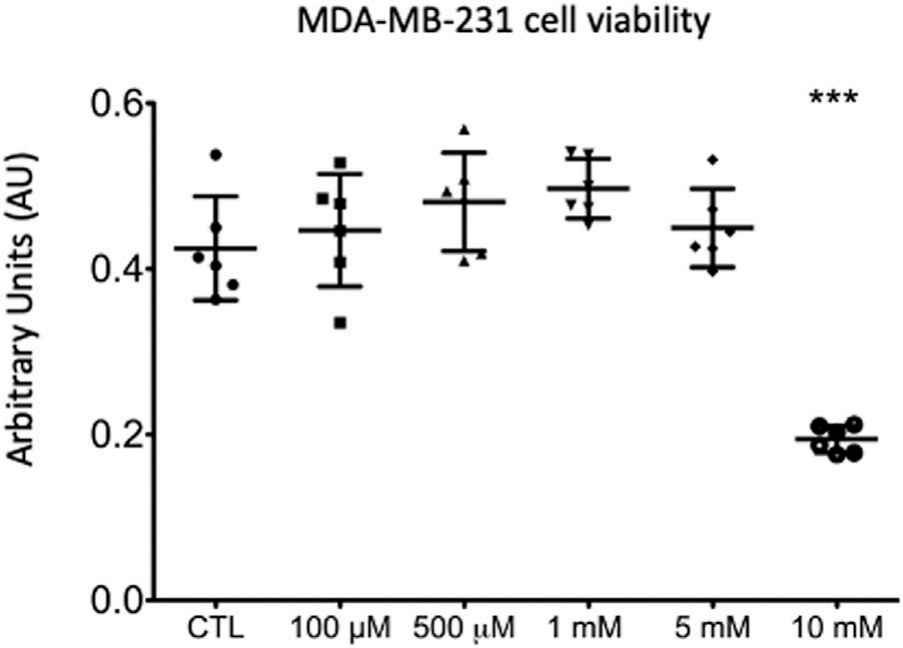
MDA-MB-231 cell viability exposed for 4 h to increased concentrations of lidocaine (from 0.01 to 10 mM) compared to cells exposed to vehicle alone (purified water, CTL). ANOVA: F (5,30) = 28.16; *p* < .0001 (***: *p* < .001).

As compared to untreated cells, MDA-MB-231 cell viability was significantly impaired when treated with lidocaine at the concentration of 10 mM (45% reduction, Figure 1) (0.194±0.016 AU versus 0.425± 0.06 AU in control group, *p* < .0001 in Dunnet test). Because of its negative effect on cell viability, the lidocaine concentration of 0.5 mM was selected for the ^1^H-HRMAS assay.

### Quality of Spectra Acquisitions

**FIGURE 2:**
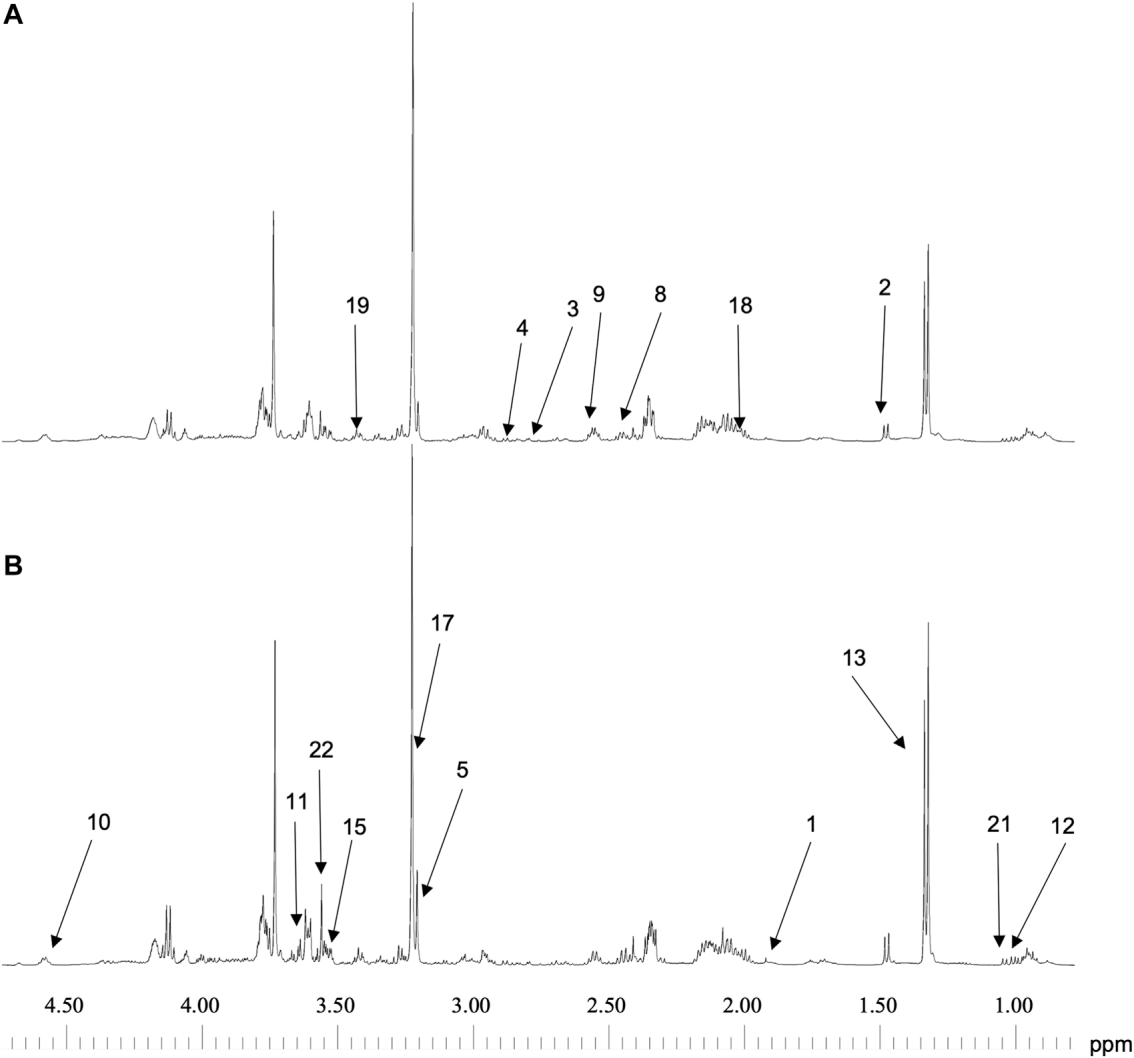
1D ^1^H CPMG HRMAS spectra of MDA-MB-231 exposed to vehicle **(A)** or to lidocaine (0.5 mM) **(B)**. Spectra can be compared because they were normalized to the sample weight. Peaks are identified as below: 1-Acetate 2-Alanine 3-Asparagine 4-Aspartate 5-Choline 8-Glutamate 9-Glutamine 10-Glutathione 11-Glycerol 12-Isoleucine 13-Lactate 15-myo-Inositol 17-Phosphocholine 18-Proline 19-Taurine 21-Valine 22-Glycine.

**FIGURE 3:**
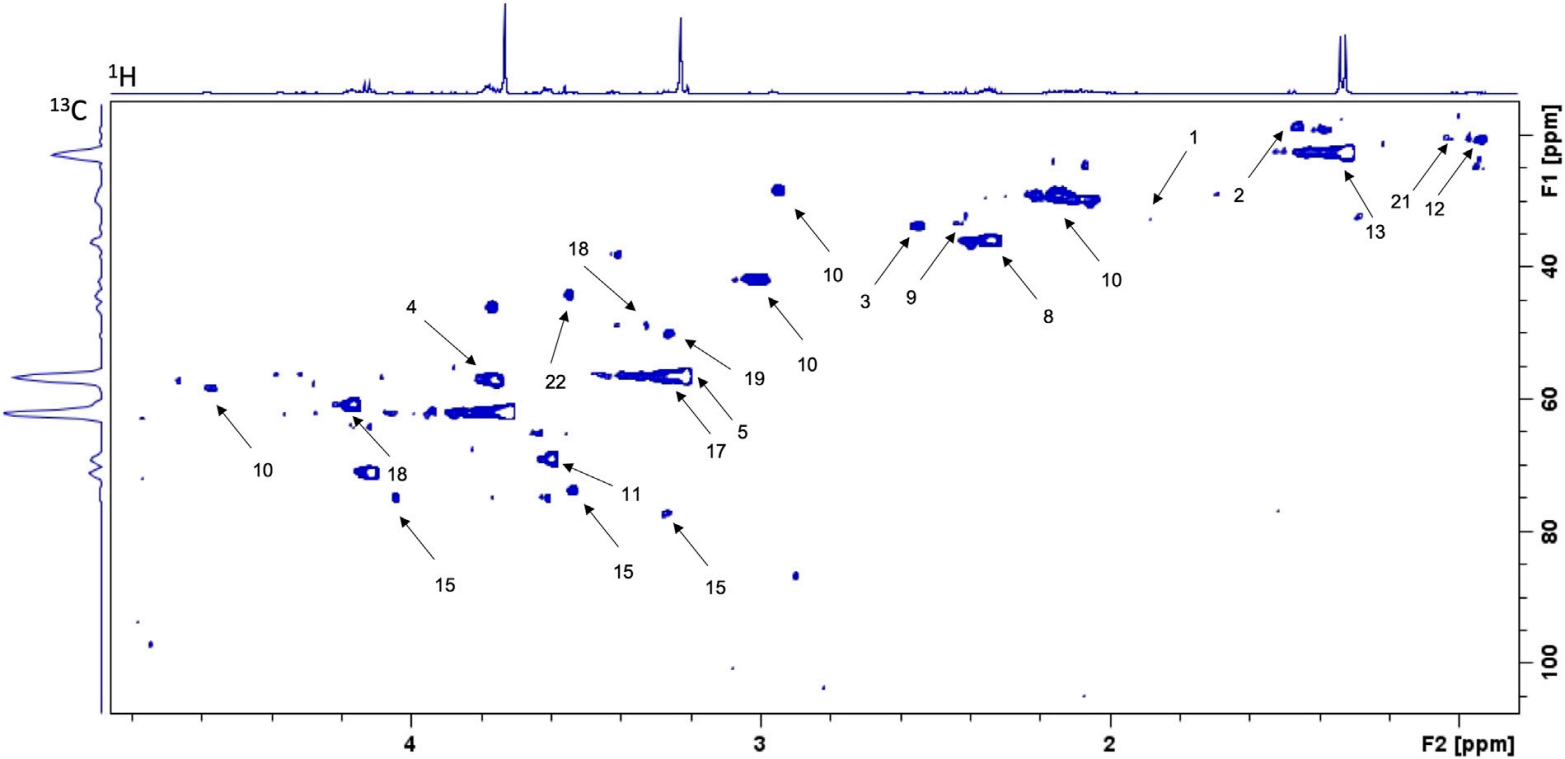
Example of 2D ^1^H-^13^C HSQC spectrum of MDA-MB-231 cells exposed to lidocaine (0.5 mM for 4 h). Spots are identified as below: 1-Acetate 2-Alanine 3-Asparagine 4-Aspartate 5-Choline 8-Glutamate 9-Glutamine 10-Glutathione 11-Glycerol 12-Isoleucine 13-Lactate 15-myo-Inositol 17-Phosphocholine 18-Proline 19-Taurine 21-Valine 22-Glycine.

Spectra of the 9 samples collected (5 for cells exposed to lidocaine and 4 for control group) were of high quality. Figure 2 represents 1D ^1^H CPMG HRMAS spectra of MDA-MB-231 cells exposed or not to lidocaine. Figure 3 represents a 2D ^1^H-^13^C HSQC spectrum of MDA-MB-231 cells exposed to lidocaine.

Twenty-two metabolites were quantified for the experiment: Alanine, Asparagine, Aspartate, Choline, Creatine, Fumarate, Glutamate, Glutamine, Glutathione, Glycerol, Isoleucine, Lactate, Malate, myo-Inositol, Phenylalanine, Phosphocholine, Proline, Taurine, Total Choline, Valine and *Glycine*. Mean values are presented in Table 1. Glucose and glycerophosphocholine were not measurable in both groups. There were no peaks of lidocaine in the samples, thus confirming efficient cell washing.

**TABLE 1:** Metabolite quantification in MDA-MB-231 cells exposed or not to lidocaine, expressed in mM [mean ± standard deviation (SD)].

	Lido *n* = 5 mean (mM)	±SD	Control *n* = 4 mean (mM)	± SD
Acetate	0,139	0,030	0,137	0,019
Alanine	0,758	0,276	0,847	0,081
Asparagine	0,727	0,264	0,828	0,196
Aspartate	0,620	0,287	0,788	0,287
Choline	0,363	0,293	0,354	0,083
Creatine	0,311	0,118	0,386	0,093
Fumarate	0,043	0,020	0,061	0,017
Glutamate	5,882	1,751	6,812	1,396
Glutamine	1,519	0,520	1,692	0,116
Reduced Glutathion	2,367	0,404	2,805	0,316
Glycerol	1,213	0,736	1,214	0,258
Isoleucine	0,260	0,081	0,242	0,027
Lactate	9,599	1,468	9,466	1,646
Malate	1,349	0,190	1,985	0,431
myo-Inositol	1,453	0,591	1,645	0,241
Phenylalanine	0,131	0,041	0,133	0,022
Phosphocholine	3,777	0,949	4,121	0,670
Proline	2,102	0,744	2,314	0,333
Taurine	1,492	0,363	1,607	0,134
TotalCholine	1,943	0,521	2,109	0,356
Valine	0,166	0,063	0,144	0,018
*Glycine*	1,086	0,605	0,985	0,089

### Lidocaine Modulates Metabolic Pathways and Decreases Cell Proliferation Potential in Triple Negative Breast Cancer Cells

**FIGURE 4:**
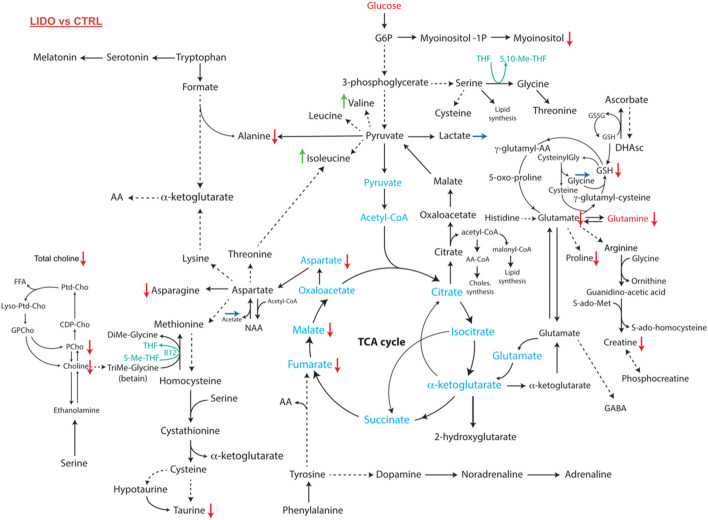
Metabolomic network of MDA-MB-231 cells exposed to 0.5 mM of lidocaine or vehicle as control for 4 h. Several pathways which promote proliferation, invasion and metastasis (glutaminolysis, choline, phosphocholine and total choline syntheses) were significantly downregulated in lidocaine group. Red, green and blue arrows indicate the decreased, the increased or the unchanged levels of metabolite after exposure to lidocaine compared to control, respectively.

Network analysis using the ADEMA algorithm shows an impairment in several metabolic pathways in MDA-MB-231 cells (Figure 4). Cell exposure to 0.5 mM of lidocaine for 4 h yielded predicted decrease in levels of metabolites involved in phospholipids metabolism and cell membrane proliferation: total choline, choline and phosphocholine. A predicted decrease in the levels of taurine, asparagine, aspartate, malate, fumarate, alanine, myoinositol, glutathione, glutamine, glutamate, proline, and creatine was also observed. On the other hand, valine, isoleucine levels were predicted to increase. Lactate, glycine and acetate levels were similar in the 2 groups (Figure 4). The metabolomic profiles indicate that lidocaine treatment of MDA-MB-231 cells at a 0.5 mM concentration impairs choline and glutaminolysis pathways and the tricarboxylic acid (TCA) cycle.

## Discussion

To our knowledge, this is the first study reporting the metabolic impact of lidocaine on the metabolomic fingerprint in cancer cells. We have demonstrated that lidocaine, at concentration of 0.5 mM for 4 h, can significantly alter metabolites levels and some metabolic pathways which are active in highly proliferative tumors.

First, our viability assay supports previous works showing a decrease in the proliferation of MDA-MB-231 cells exposed to lidocaine (Chamaraux-Tran and Piegeler, 2017; Chamaraux-Tran et al., 2018; D’Agostino et al., 2018). It was mandatory for us to determine a lidocaine concentration with no significant effect on cell viability to have the same quantity of cells for the HR-MAS NMR assay. In this experiment we used lidocaine hydrochloride monohydrate to avoid absolute ethanol as solvent which might compromise NMR assay. As higher concentrations of lidocaine hydrochloride monohydrate were needed to decrease cell viability [10 versus 0.5 mM of lidocaine prepared in absolute ethanol (Chamaraux-Tran et al., 2018)], it confirms that excipient may have a direct antitumor effect (Chamaraux-Tran and Beloeil, 2018). To note, higher concentrations of lidocaine (10 mM) were needed in our experiment compared to previous studies on MDA-MB-231 cells, independently to solvent. Jiang et al. (2016) and Li et al. (2018) have demonstrated that lidocaine from 1 mM was able to significantly decreased cell viability in a concentration-dependent manner. These results could be explained by shorter exposure in our study (4 h versus 24 or 48 h, respectively).

The HR-MAS NMR assay finds similar metabolomic fingerprints for MDA-MB-231 cells to a previous work (Maria et al., 2015). To note, glucose was not measurable in both groups due to the highly intense glucose uptake and glycolysis in most solid tumors compared to normal tissues. High levels of choline-rich metabolites are mainly due to increased phospholipid turnover and cell membrane synthesis in proliferative cells. Intense glutaminolysis promotes tumor proliferation and chemoresistance, in part through activation of the PI3K/AKT/mTORC1 pathway (Vignoli et al., 2021). Lidocaine causes a decrease in the metabolites of these two pathways, which reflects its impact on the proliferative potential of cancer cells. These results confirm previous experiments on lung cancer (Sun and Sun, 2019) and hepatocellular carcinoma cells (Zhang et al., 2020) showing an inhibitor effect of lidocaine on PI3K/AKT/mTORC1 pathway, evidenced by assessing the phosphorylation levels of PI3K and AKT by western blot. The metabolomic impact of lidocaine choline pathway is comparable to the effects of some chemotherapies on this triple negative cell line: Maria et al. (2017) have demonstrated that cisplatin and tamoxifen could significantly reduce phosphocholine content suggesting a direct antiproliferative effect.

The tricarboxylic cycle (Krebs cycle) of MDA-MB-231 cells is also affected by lidocaine. There is a decrease in fumarate, malate and alanine. For instance, fumarate inhibits prolyl-hydroxylases, which leads to an increase in HIF-1α levels and allows, among other things, the survival of cancer cells exposed to hypoxia (Kuo et al., 2016). Thus, lidocaine could modulate the HIF-1-induced proliferation pathway as it was suggested in other studies. Indeed, western blot and/or gene expression experiments showed that lidocaine impairs HIF-1 pathway in renal and neuronal cells (Okamoto et al., 2017) or in human hepatoma and neuroblastoma cell lines (Nishi et al., 2005).

Our results showed a decrease in glutathione in its reduced form (GSH), which could be linked to the decrease in myoinositol. The level of glutamate, which is a precursor of GSH, is lowered; its synthesis may thus also be compromised by lidocaine. Another likely hypothesis would be glutathione consumption in response to increased oxidative stress. Indeed, a previous work investigating the impact of lidocaine on yeast cells observed an initial decline in GSH at H+1 but a gradual increase in this antioxidant from H+2, which may be a counter-regulation mechanism against oxidative stress induced by lidocaine (Boone et al., 2017). Similarly, an *in vitro* study showed that lidocaine caused a decrease in mitochondrial membrane potential and an increase in free radical production in non-small cell lung cancer cells (Wang et al., 2016). Furthermore, the absence of glucose and glycerophosphocholine in our samples and the similar levels of lactates in the control and lidocaine groups indicate that the glycolysis pathway and the choline pathway remain highly active in MDA-MB-231 cells despite lidocaine treatment. Indeed the increase of glycolysis in the tumor cell is a well-known phenomenon described as the Warburg effect (Wishart et al., 2016). It is linked to tumor overexpression of glucose membrane transporters, to the increase in hexokinase activity involved in glucose phosphorylation, and to the increase in anaerobic cellular glycolysis by inhibition of the oxidative pathway. Similarly, while glycerophosphocholine (GPC) levels still cannot be measured under lidocaine treatment, the fact that phosphorylcholine (PC) levels are lowered supports an increase in the GPC/PC ratio, which is considered a good prognosis factor in a cohort study in patients with *in situ* root canal carcinoma biopsies (Chae et al., 2016) and in a cohort of patients with gliomas (Dali-Youcef et al., 2015) or oligodendroglioma (Bund et al., 2019).

The effects of decreased myoinositol by lidocaine should be further investigated. In fact, inositols have important antiproliferative properties (Bizzarri et al., 2016). For instance, they can interfere with cell proliferation by decreasing the PI3K level or inhibiting pRB phosphorylation or Akt activation and therefore NF-kB. They can also interfere with cell invasion and the epithelial-mesenchymal transition (Bizzarri et al., 2016).

We have shown here that lidocaine at a concentration of 0.5 mM for 4 h can modulate the metabolism of triple negative cells. This concentration is compatible with clinical use of lidocaine infiltration for local anesthesia as lidocaine is frequently used at the concentration of 10 mg/ml (=42 mM, MW = 234,34 g·mol−1) but not with systemic intravenous administration. Indeed, over a plasma concentration of 21 μg/ml (90 μM) (Beaussier et al., 2018), patients may experience the systemic toxicity of lidocaine with neurologic symptoms ranging from cognitive disorders to seizures and cardiovascular compromise ranging from rhythm disorders to cardiac arrest. Moreover, lidocaine plasma levels following its intravenous administration are in a range of 1,4–6 μg/ml (25 μM) (Beaussier et al., 2018).

Therefore, the deepening of our study, investigating dose-effects and time-effects curves, would allow us to determine the molecular mechanisms at play and potential clinical use given the antitumoral properties of lidocaine. Similarly, *in vitro* studies in breast cancer (Li et al., 2014) and *in vivo* findings in hepatocellular carcinoma (Xing et al., 2017) showed that lidocaine can have a synergistic effect with cisplatin. It would thus be worth studying these combined effects in metabolomics to reach lidocaine doses below its toxic thresholds allowing its systemic use and repositioning lidocaine in chemotherapy.

Finally, as metabolomic profiles in oncology are established to develop prognostic strategies capable of classifying different breast cancers or therapeutic strategies in personalized medicine, it appears important to continue this work. Thus, studying the impact of lidocaine on an *in vivo* model (such as PDX xenograft) would get closer to physiological conditions, that could be transposed into the clinical arena. Indeed, if the cell culture allows for a simple experimental approach, both the nutritional conditions (excess glucose in the culture medium) and the oxygen concentrations (ranging from hyperoxia to hypoxia in some parts of the flask if it is not agitated) often do not allow extrapolation of experimental results to clinical use. Moreover, our *in vitro* study doesn’t assess the impact of lidocaine on the microenvironment while it has been shown that local anesthetics could affect viability and differentiation capacity of adult stem/progenitor cells (Kim et al., 2020; Kubrova et al., 2021). Those effects on mesenchymal stem cells could influence wound healing or tumor spreading after surgery (Lucchinetti et al., 2012). Similarly, the study should be extended to other anesthesia drugs (hypnotics and analgesics in particular) that may also affect tumor progression (Sekandarzad et al., 2017). It would allow for a standardization of tumor sampling protocols in breast cancer surgery. Indeed, if percutaneous biopsies and clips are systematically performed under local anesthesia by lidocaine, the dose administered is not standardized. Similarly, wire localization by ultrasound before surgical excision can be done the day before or the morning of the surgery and the local anesthesia is not systematic. It depends for example on the expected difficulties, the anatomic structures that will be crossed, the patient’s wish or the type of localization device. Finally, in the context of multimodal analgesia during surgery, patients can benefit from regional anesthesia (plane or paravertebral blocks) or intravenous lidocaine administration. Similarly, *in vivo* studies have shown that other analgesic drugs such as morphine or hypnotics needed for general anesthesia can affect cellular metabolism (Sonnay et al., 2017). Hu *et al.* have recently shown that propofol, the most commonly used anesthetic drug, could alter metabolism of lung cancer cells (Hu et al., 2021). In this study, propofol increased intracellular glutamate and glycine but decreased acetate and formate in A549 cell line. Considering the results of this study and our own, it would be interesting to find a protocol which could have a direct protective effect against circulating cells or micro-metastasis, which development may be favored during the perioperative time (Benish and Ben-Eliyahu, 2010). All those parameters should also be considered to establish metabolomic fingerprints.

## Conclusion

Our *in vitro* study showed that, under our experimental conditions, lidocaine at clinical concentrations useful for surgical site infiltration inhibits the proliferation of a high dose triple negative breast cancer cell line. At lidocaine concentrations that do not affect cell viability *a priori*, there is an inhibition of several overactive metabolic pathways in oncogenesis. This effect could have interesting clinical applications in several respects: 1) for local tumor recurrence, lidocaine may prevent the proliferation of a possible remnant of malignant cells at the surgical site; 2) for metastases, this local anesthetic may limit the spread of tumor cells.

On the other hand, the concentrations studied in our work were higher than systemic toxic thresholds. Further works are needed to refine the dose-response relationship of the observed effects and possibly to find a synergistic effect with conventional antiproliferative drugs. Our experimental results will need to be supplemented and tested in prospective multi-year clinical studies using either infiltration or intravenous analgesia. Our *in vitro* data are also interesting because they are part of the current trend of over-specialization in «onco-anesthesia». In this context, anesthesiologists should be made aware of the impact of their management as specialists in perioperative medicine on the long-term oncological outcomes of patients anesthetized for cancer surgery. Additionnally the impact of local anesthetics should be considered to establish metabolomic fingerprints in cancer.

## Data Availability

The original contributions presented in the study are included in the article/Supplementary Material, further inquiries can be directed to the corresponding author.
